# Umbilical cord tissue is a robust source for mesenchymal stem cells with enhanced myogenic differentiation potential compared to cord blood

**DOI:** 10.1038/s41598-020-75102-9

**Published:** 2020-11-04

**Authors:** Shivangi Mishra, Jayesh Kumar Sevak, Anamica Das, G. Aneeshkumar Arimbasseri, Shinjini Bhatnagar, Suchitra D. Gopinath

**Affiliations:** 1 Pediatric Biology Center, Translational Health Science and Technology Institute (THSTI), NCR Biotech Science Cluster, 3rd Milestone, Faridabad-Gurgaon Expressway, PO Box #04, Faridabad, 121001 India; 2 National Institute of Immunology, Aruna Asaf Ali Marg, New Delhi, India

**Keywords:** Molecular biology, Stem cells

## Abstract

Differentiation of mesenchymal stem cells (MSCs) derived from two different sources of fetal tissues such as umbilical cord blood (UCB) and tissue (UCT) into skeletal muscle have remained underexplored. Here, we present a comparative analysis of UCB and UCT MSCs, in terms of surface markers, proliferation and senescence marker expression. We find that CD45^−^CD34^−^ MSCs obtained from UCT and UCB of term births display differences in the combinatorial expression of key MSC markers CD105 and CD90. Importantly, UCT MSCs display greater yield, higher purity, shorter culture time, and lower rates of senescence in culture compared to UCB MSCs. Using a robust myogenic differentiation protocol, we show that UCT MSCs differentiate more robustly into muscle than UCB MSCs by transcriptomic sequencing and specific myogenic markers. Functional assays reveal that CD90, and not CD105 expression promotes myogenic differentiation in MSCs and could explain the enhanced myogenic potential of UCT MSCs. These results suggest that in comparison to large volumes of UCB that are routinely used to obtain MSCs and with limited success, UCT is a more reliable, robust, and convenient source of MSCs to derive cells of the myogenic lineage for both therapeutic purposes and increasing our understanding of developmental processes.

## Introduction

Mesenchymal stem cell (MSC)-based cellular therapies rely on the clonogenic, proliferative, genetically stable, differentiation ability into the three germ layers, and immunological privileged state exhibited by MSCs^1,2^. This has led to several studies exploring sources of MSCs that represent a robust reservoir, convenient to isolate, and exhibiting potent differentiation potential^3^. In this regard, isolation of MSCs from human umbilical cord and its various compartments has emerged as a compelling alternative because of its ease in harvesting, relatively greater stem cell numbers, and increased stemness potential^4,5^. Recent studies have also explored the use of umbilical cord-derived MSCs (uMSCs) as a model to investigate metabolic and gene expression differences underlying fetal body composition in relation to specific maternal parameters^6,7^. For instance, MSCs of infants exposed to maternal obesity displayed alterations in lipid metabolism when induced to undergo adipogenesis and was reflective of higher infant fat mass^6,8^. While the differentiation of uMSCs into adipocytes, osteocytes, and chondrocytes is a well-documented phenomenon and defines the multipotential nature of MSCs, very few studies have explored the differentiation of uMSCs into the skeletal muscle lineage^9,10^. More importantly, a comprehensive comparative characterization of myogenic differentiation potentials between MSCs from different compartments of the umbilical cord is lacking. This has been in part due to inconsistent rates in establishing successful cultures of MSCs from UCB specifically from term births and conflicting results on the volumes of cord blood required to be collected for obtaining sufficient numbers of MSCs^4,11–13^.

Postnatal skeletal muscle differentiation progresses by myogenic progenitor cells called “satellite cells” expressing the paired box protein Pax7, followed by a multi-step process coordinated by a series of transcription factors, Myf5, MyoD, Myogenin, and MRF4 that are expressed sequentially to regulate lineage determination, fusion and terminal differentiation^14,15^. In order to recapitulate the full program of myogenesis in vitro from pluripotent or multipotent cells, it becomes imperative to document the expression of hallmarks of myogenic progression, thereby ensuring definitive lineage commitment and progression. Earlier studies report that MSCs can be induced to differentiate into the myogenic lineage by treatment with 5-Azacytidine and transfection of MyoD^16,17^. However, the external introduction of epigenetic reprogramming in these processes can alter existing chromatin marks on the fetal epigenomic landscape, thereby confounding epigenetic signatures resulting from intrauterine programming. Induction of myogenic differentiation was also reported in UCB MSCs using a combinatorial steroidal treatment^6,18^. However, in all these studies, a comprehensive characterisation of their conversion to the myogenic lineage or consistency in yield from different fetal tissue compartments remains to be studied. In this study, we report that term birth UCT MSCs differ from UCB MSCs in the combinatorial expression of MSC markers, proliferation capacity and senescence rates. Using a reliable and robust method to convert uMSCs to skeletal muscle, we report paired data from the same neonate showing that UCT MSCs possess a more robust myogenic potential than UCB MSCs as evidenced by transcriptomic profiling and specific marker analysis. Finally, functional assays demonstrate a necessary requirement for CD90 expression as a key factor in promoting myogenic differentiation in uMSCs, thereby accounting for the superior myogenic potential of UCT over UCB MSCs.

## Results

### UCT and UCB MSCs differ in key combined surface marker expression, proliferation and senescence characteristics

**Figure 1:**
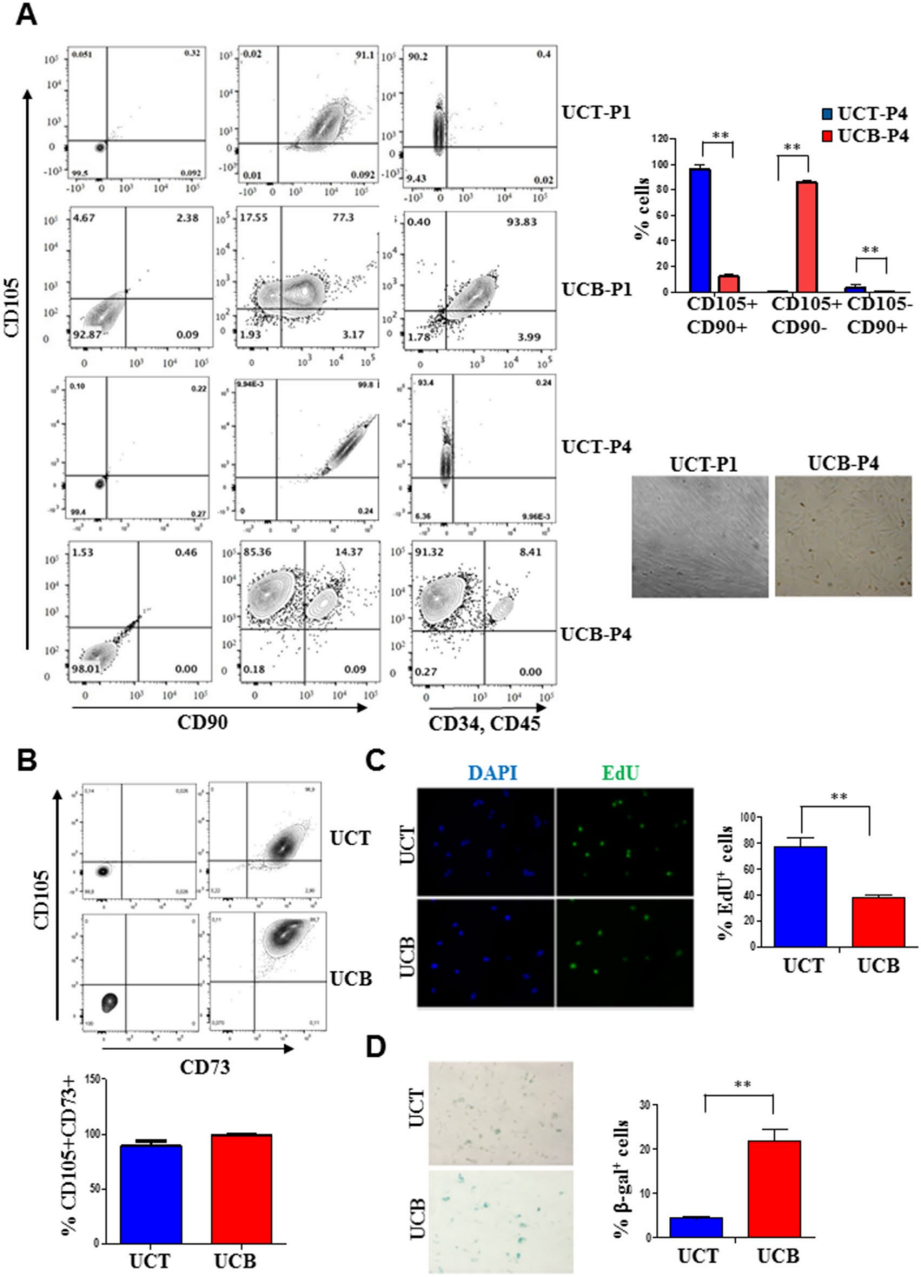
Comparative analysis of MSC marker expression in UCB and UCT. (**A**) UCT-P1 MSCs display greater expression of MSC markers in ~ 90% of analysed cells (*first row panels*) compared to UCB-P1 MSCs that continue to express hematopoietic markers *(second row panels*). At P4, CD34^−^CD45^−^ UCT MSCs express CD105^+^CD90^+^ (third row panels) compared to UCB MSCs that are predominantly CD105^+^CD90^−^ (*last row panels,* graph). Phase contrast images of UCT and UCB-P1 MSCs show difference in morphologies upon isolation (**B**) Coexpression of CD73 and CD105 in UCT and UCB MSCs at P4. UCT MSCs display greater proliferation as seen in (**C**) EdU incorporation assays of UCT and UCB MSCs at P4, and lower senescence rates as seen in (**D**) Senescence-associated β-galactosidase (SAβG) staining in UCT and UCB MSCs. (For all figures N = 5, n = 3,**p = 0.002).

To determine the expression of key surface markers of MSCs derived from UCT and UCB, we evaluated combinatorial expression patterns of CD105, CD90, and CD73. We found that the combined expression pattern of CD105 and CD90 was different between UCT and UCB MSCs. Specifically, we found that most of the UCT MSCs upon immediate isolation (UCT-P1) were CD105^+^CD90^+^ and devoid of cells of hematopoietic origin characterised by their CD45^−^CD34^−^ status (Fig. 1A, first row of panels, N = 5). On the other hand, UCB MSCs after immediate isolation in the first passage (UCB-P1) that were CD105^+^CD90^+^ were also CD45^+^CD34^+^ (Fig. 1A, second row of panels, N = 5). Only after repeated passaging of UCB MSCs (for 3 passages) was there a huge reduction in the CD45^+^CD34^+^ population. However, at this point UCB MSCs were predominantly CD105^+^ with only a smaller population that was CD105^+^CD90^+^ (Fig. 1A, last row of panels, N = 5). UCT MSCs meanwhile continued to maintain their phenotype at later passages (Fig. 1A, third row of panels, graph to the right shows quantitation of populations in UCT and UCB at P4, N = 5) The morphologies of the UCT and UCB MSCs at passages that were devoid of contaminating hematopoietic cells showed that while UCT MSCs were spindle-shaped, long and aligned in one axis, UCB MSCs were more fibroblast-like, stellate and spread out (Fig. 1A, right lower panel). Unlike differential CD105 and CD90 co-expression, no differences were observed with respect to the co-expression of another key MSC marker, CD73 between UCT and UCB MSCs (Fig. 1B and graph below). It is noteworthy that of the 5 biological replicates used in the study, 3 of the neonates provided paired samples for both UCB and UCT derived MSCs.

To investigate the cell cycle characteristics of UCB and UCT MSCs, we determined the number of *S-phase* cells and senescence characteristics in these cultures at the same passages. Upon pulse-labeling cultures with 5-ethynyl-2′-deoxyuridine (EdU) we found that after 6 h, UCT MSCs displayed twice the number of EdU^+^ cells compared to UCB MSCs (Fig. 1C, graph on the right, N = 5). Importantly, UCB MSCs displayed higher rates of senescence as evidenced by the expression of β-galactosidase expression compared to UCT MSCs at the same passage (Fig. 1D, graph on the right, N = 5). Thus, compared to UCB, UCT-MSCs not only display differential combined surface marker expression, but also serve as a more robust source of MSCs that display higher proliferation and lower senescence rates.

### Chemical induction and adhesion modulation convert UC MSCs into skeletal myogenic lineage

**Figure 2:**
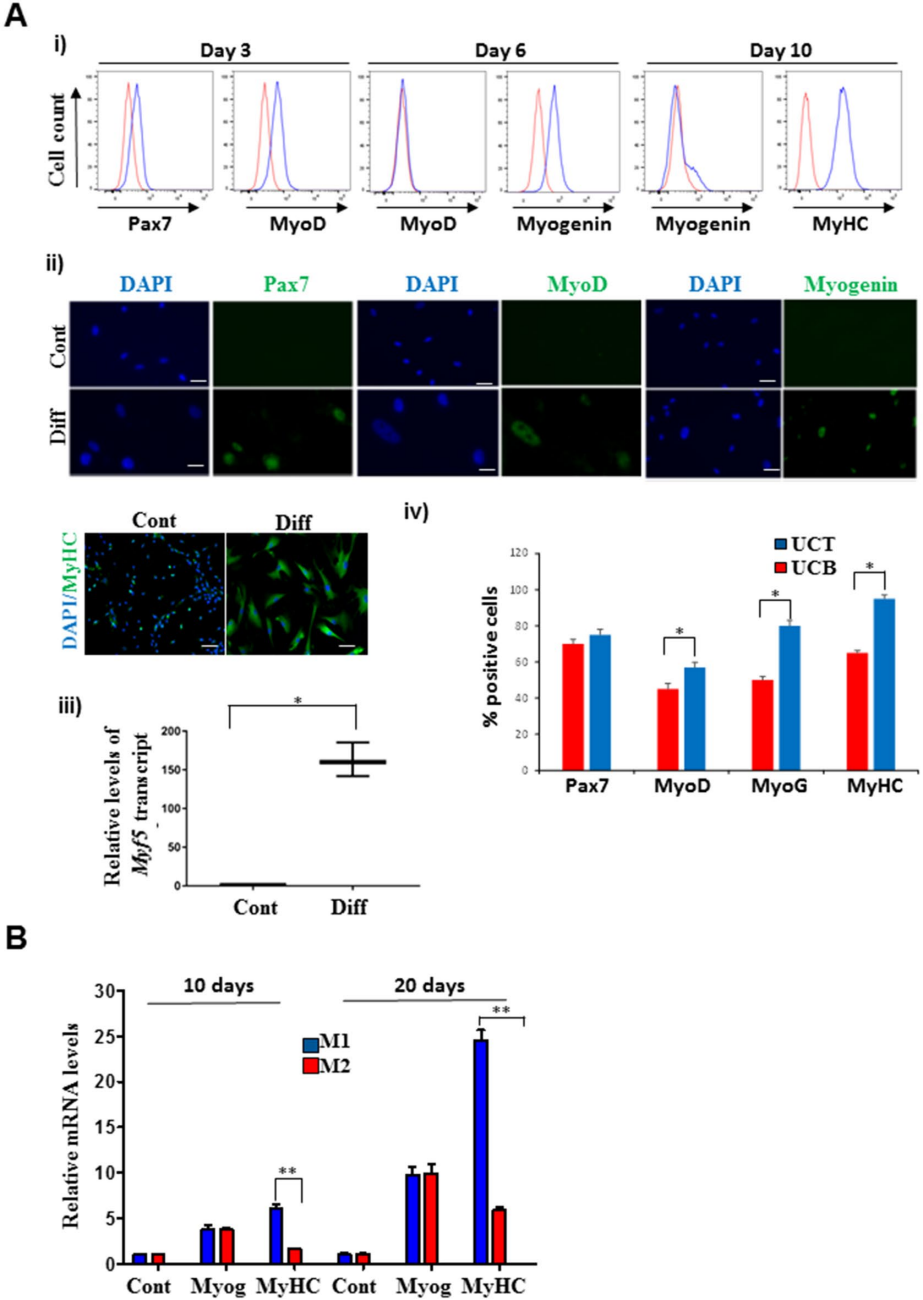
Conversion of uMSCs into myogenic lineage. (**A**) Myogenic differentiation was induced by culturing UCB MSCs in myogenic differentiation medium M1 for 3, 6, and 10 days to detect Pax7, MyoD, Myogenin, and MyHC by (**i**) FACS and by (**ii**) immunocytochemistry. Nuclei were counterstained with DAPI. (**iii**) Myf5 mRNA expression in control and differentiated UCB MSCs after 3 days (*p = 0.03). (**iv**) Comparative immunofluorescent analysis of Pax7 and MyoD at 3 days, Myogenin at 7 days and MyHC at 10 days between UCT and UCB MSCs. UCT MSCs show increased expression of MyoD, Myogenin, and MyHC expressing cells compared to UCB MSCs at their corresponding time points (*p = 0.03). (**B**) Myogenin and MyHC mRNA expression in control UCT MSCs or UCT MSCs induced to differentiate in M1 medium with enhanced adhesion or M2 medium for 10 and 20 days (**p = 0.002). Robust expression of MyHC was observed in M1 medium with enhanced adhesion at 10 and 20 days.

To optimize conditions for skeletal myogenic differentiation of MSCs, we developed a modified protocol and conducted a detailed examination of myogenic progression. Both UCT and UCB MSCs were induced to differentiate in differentiation medium (M1) in the presence of enhanced adhesion with laminin and collagen. To ensure that myogenic differentiation was indeed occurring in the uMSCs, we initially used UCB MSCs as a model system because of its usage in earlier protocols^18^. A representative analysis of myogenic gene expression in differentiated UCB MSCs at 3, 6, and 10 days during myogenesis using flow cytometry and immunofluorescence shows the profile of transcription factors present in muscle stem cells (satellite cells) such as Pax7, MyoD at 3 days, Myogenin at 6 days and Myosin heavy chain (MyHC) at 10 days after induction of myogenic differentiation (Fig. 2A i, ii). Consistent with their sequential expression during myogenic progression, we observed from FACS analysis that MyoD expression that could be detected at 3 days declined at 6 days, while Myogenin expression detected at 6 days declined by 10 days of differentiation, recapitulating their order of expression during myogenesis (Fig. 2A, i). From the immunofluorescent analysis, we observed that differentiated MSCs, particularly in the later time points possessed larger nuclei and increase in cytoplasmic volume (Fig. 2A, ii). *Myf5* mRNA could also be detected at 3 days after MSCs were induced to differentiate (Fig. 2A, iii). As a control group, we used a myogenic cell line, C2C12 to detect myogenic proteins (Supplementary Fig. S1).

A comparative analysis of myogenic differentiation between UCT and UCB MSCs revealed that UCT MSCs express greater numbers of MyoD, Myogenin, and MyHC expressing cells than UCB MSCs (Fig. 2A, iv). Further analysis of myogenic capacities revealed that MyHC positive myotubes in UCT and UCB MSCs exhibited different morphologies (Supplementary Fig. S1). In UCT MSCs that were differentiated into the myogenic lineage for 15 days we observed cells with longitudinal morphology with nuclei arranged along the longitudinal axis similar to the behaviour of bonafide myogenic cells. However, with UCB MSCs differentiated under the same conditions for the same length of time, we observed nuclei in disorganized sacs similar to “myosacs” (Supplementary Fig. S1).

We compared our protocol for induction of myogenic differentiation with one that was used to differentiate mouse and human pluripotent stem cells into the myogenic lineage in UCT MSCs since they exhibited more robust myogenic differentiation capacities^19^. We found that as early as 10 days, we could detect the expression of *MyHC* in MSC cultures in our media (M1) compared to the media used for embryonic stem cells (M2) (Fig. 2B). This difference in *MyHC* expression persisted till 20 days after induction of differentiation. While *Myogenin* expression was also induced both in M1 and M2, there were no differences in the pattern of expression of *Myogenin* between the two media for the duration of the experiment (Fig. 2B). These results suggest that our protocol of using M1 media and enhanced adhesion is a more robust inducer of myogenic differentiation in MSCs.

### Tissue-specific-transcriptomic analysis reveal an upregulation of several skeletal muscle-elevated genes and differential myogenic potentials between UCT and UCB MSCs

**Figure 3:**
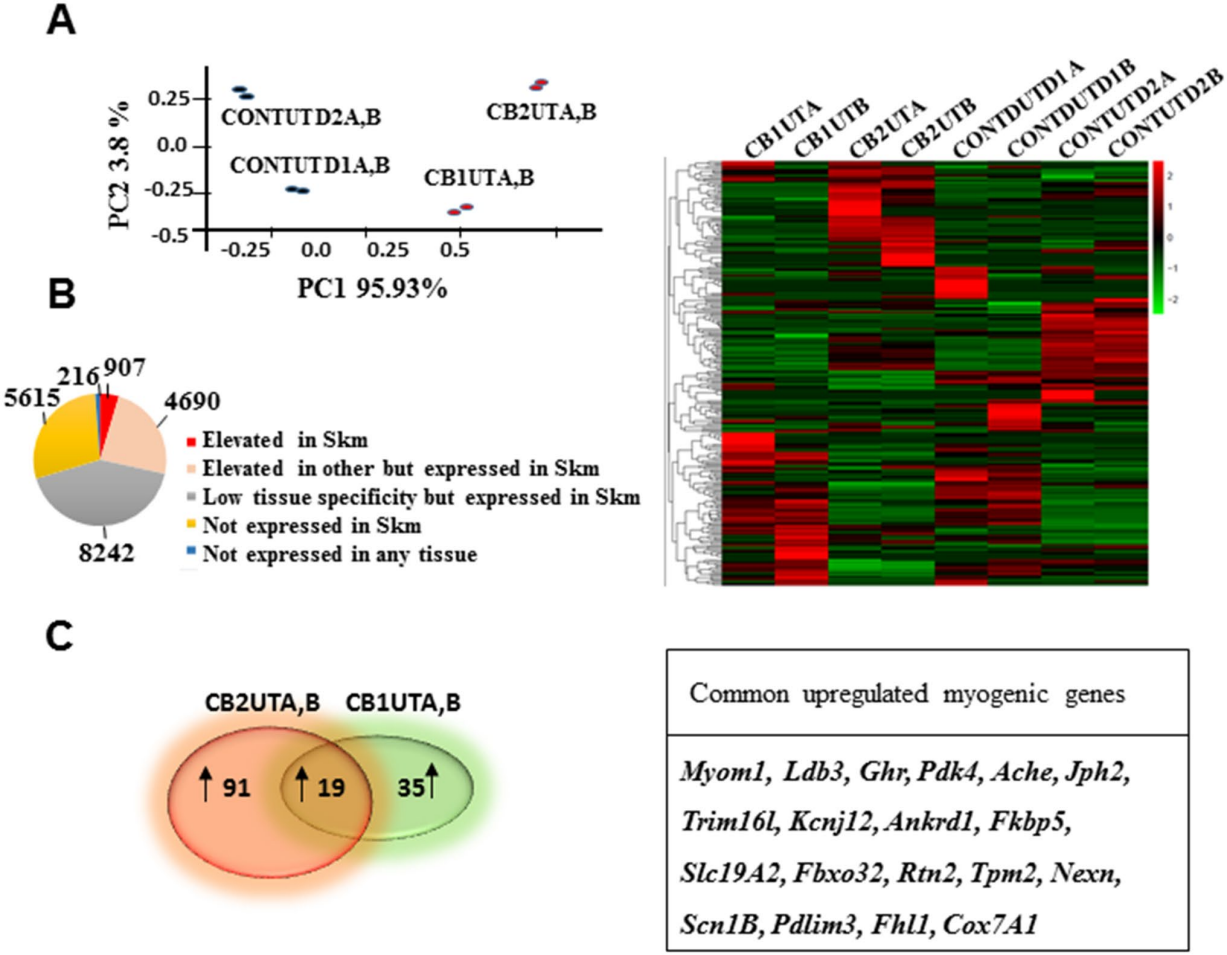
Transcriptomic profiling of uMSCs converted to the myogenic lineage. (**A**) PCA of untreated MSCs and MSCs induced to differentiate for 7 days using all genes. (**B**) Pie chart showing distribution of skeletal muscle-specific genes (red slice, 907 genes). Heat map of normalized counts of 907 genes between control MSCs and MSCs differentiated for 7 days. (**C**) Venn diagram showing upregulated myogenic genes in UCT compared to UCB (log_2_ fold change ≥ 1 and a *p*Adjusted value ≤ 0.05). Table shows common upregulated myogenic genes between UCT and UCB MSCs.

To investigate the extent of myogenic differentiation in UC MSCs with our modified protocol, we performed transcriptomic profiling of MSCs from UCB (CONTUTD1) and UCT (CONTUTD2) that were differentiated for a week (CB1UT and CB2UT respectively) and were derived from the same neonate. Principal component analysis (PCA) using the whole transcriptome shows that undifferentiated MSCs from the two sources (CONTUTD1, CONTUTD2) and their differentiated MSC counterparts (CB1UT and CB2UT respectively) cluster into distinct groups, with technical replicates (A, B) for each sample clustering together (Fig. 3A). Of the list of genes that were altered, we queried for changes in gene expression of 888 genes represented in the TruSeq RNA library kit from a list of 907 genes that are elevated in skeletal muscle in comparison to other tissues (https://www.proteinatlas.org/humanproteome/tissue/skeletal+muscle#the_skeletal_muscle_transcriptome) in response to induction of myogenic differentiation in MSCs (Fig. 3B). This list includes “tissue enriched”, “group enriched”, and “tissue enhanced” gene expression which are quantitatively higher in skeletal muscle compared to other tissues. The skeletal muscle-specific heat map revealed that MSCs from both sources, CONTUTD1 and CONTUTD2 lines displayed changes in myogenic gene expression upon induction of differentiation after normalization to their respective controls (*p* < 0.05 and log2 fold change ≥ 1) (Fig. 3B). When we compared the number of upregulated myogenic genes in their respective differentiated lines, CB1UT AND CB2UT, we found several common myogenic genes (19 genes) as well as genes that were unique to CB1UT (35 genes) and CB2UT (91 genes) (Fig. 3C). Of the common genes, tissue-specific gene ontology analysis using the PANTHER GO-slim database identified 2 major protein classes such as cytoskeletal proteins associated with actin binding and sarcomere assembly (*TPM2, LDB3, PDLIM3, FHL1, NEXN, MYOM1*) and transporters associated with contractile function (*RTN2, SLC19A2, ACHE, SCN1B, SLC19A2, JPH2*, *KCNJ12, ANKRD1*) in skeletal muscle. Additionally, proteins associated with muscle mass maintenance (*FBXO32, TRIM16L, GHR*), calcium signalling (*FKBP5*), and enzymatic function (*COX7A1, PDK4*) were also present just within a week after induction of myogenic differentiation of MSCs (Fig. 3C).

### UCT MSCs display more robust myogenic potential compared to UCB MSCs

**Figure 4:**
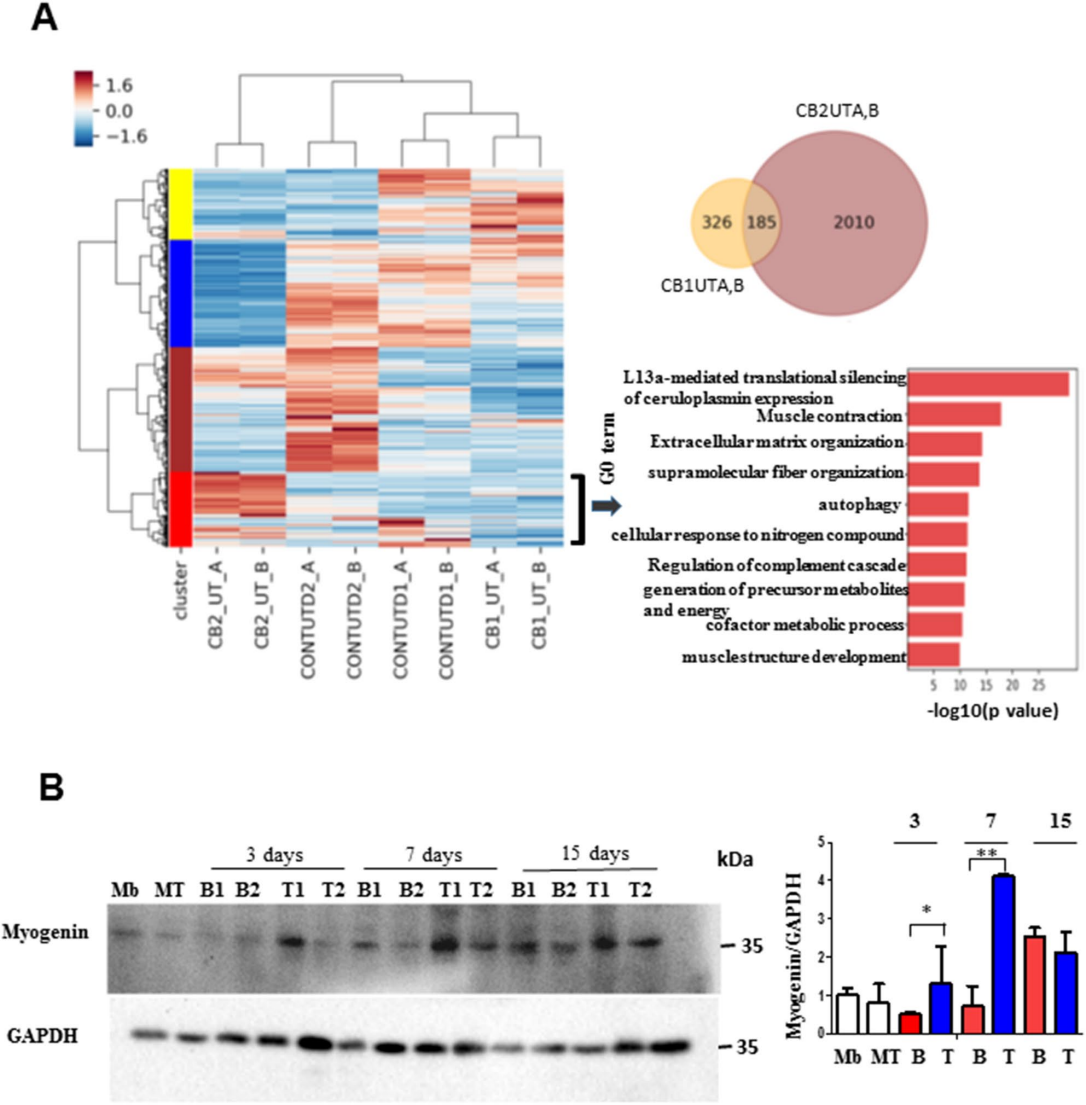
UCT MSCs display robust myogenic potential compared to UCB MSCs (**A**) Heat map of normalised counts for genes from skeletal muscle proteome in UCT MSCs and UCB MSCs. Venn diagram of upregulated genes in UCT versus UCB MSCs after 7 days of myogenic differentiation. Metascape analysis of the red cluster from heat map (*p* < 0.05). (**B**) Representative western blot of lysates from proliferating myoblasts (Mb), myotubes (MT), UCB and UCT MSCs induced to differentiate for 3, 7, 15 days, and probed with Myogenin antibody. (**p = 0.001, *p = 0.043).

Consistent with the increased number of myogenic genes induced upon differentiation of UCT MSCs (CB2UT) from a list of genes elevated in skeletal muscle tissue, we compared myogenic gene expression from differentiated UCB and UCT MSCs with a catalogue of genes corresponding to 5431 non-redundant skeletal muscle proteins compiled by Gonzalez-Frier et al.^20^. This list was created from peer-reviewed publications spanning 2002-November 2015 identified by mass-spectrometry based proteomics selectively for establishing the human skeletal muscle proteome. As observed with the skeletal muscle-elevated gene list, we observed that UCT MSCs displayed a greater number of myogenic genes (CB2UT, 2010 genes) that were upregulated compared to UCB MSCs from the same neonate (CB1UT, 326 genes) (Fig. 4A). A clustered heat map analysis of the normalised read counts for these genes in these samples revealed 4 different clusters (Fig. 4A, red, brown, blue, and yellow) that display different trends in myogenic gene regulation between UCT and UCB-derived MSCs. Genes in the red cluster that appear to be upregulated in differentiated UCT MSCs, while relatively unchanged in differentiated UCB MSCs, were enriched for GO annotations associated with terminal myogenic genes related to contractility, muscle structure development, protein translational control, autophagy, and extracellular matrix organization functions, all of which are critical for terminal muscle differentiation and function (Fig. 4A, bottom right).

Kinetic analysis of expression of a specific myogenic gene, *Myogenin* of UCB and UCT MSCs over the course of 15 days revealed that within 7 days of induction of myogenic differentiation, Myogenin was robustly expressed in UCT MSCs compared to UCB MSCs (Fig. 4B, graph, full length blots provided in Supplementary Fig. S2).

### CD90 expression promotes enhanced myogenic capacity in UCT MSCs

**Figure 5:**
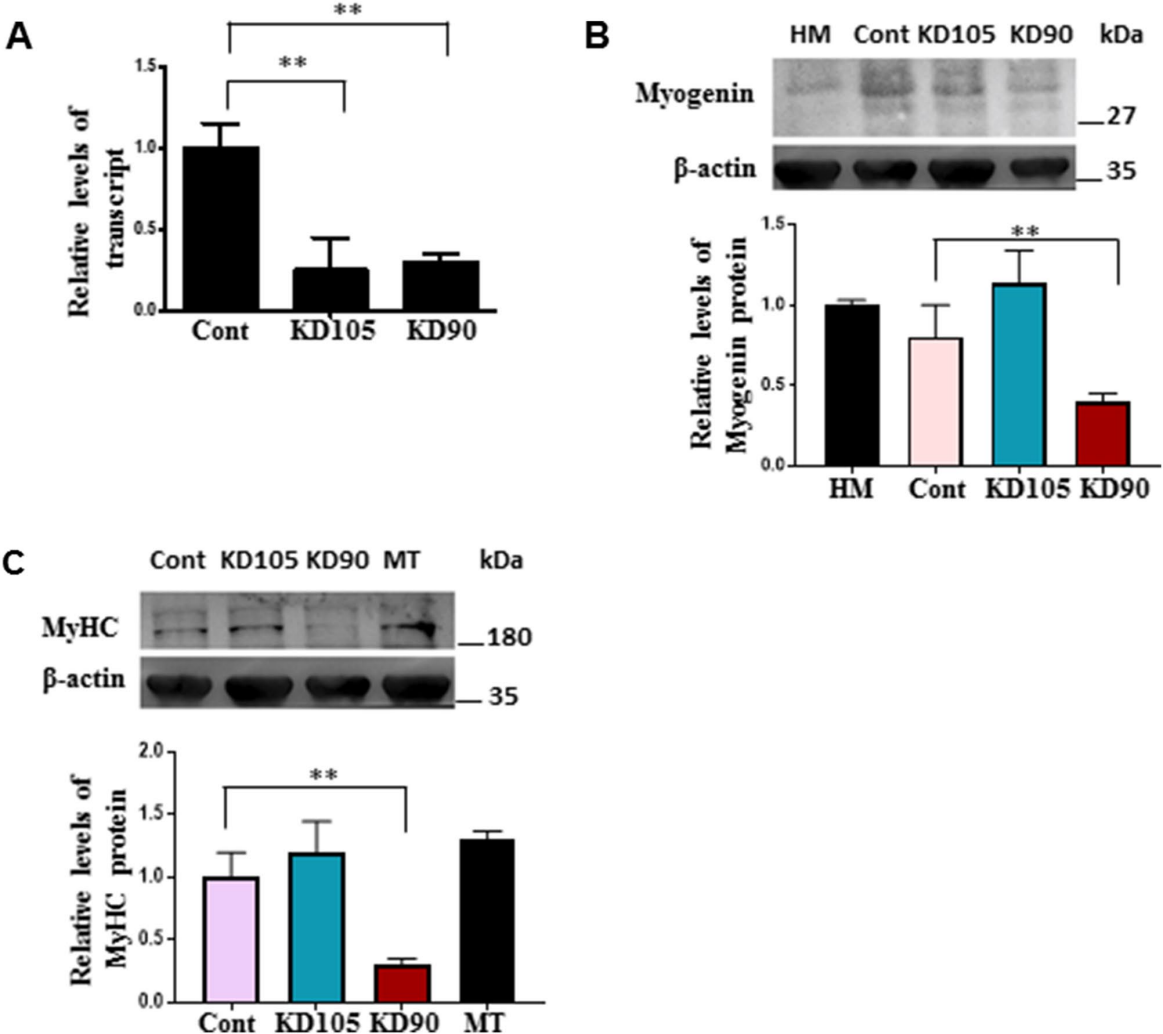
CD90 expression promotes enhanced myogenic differentiation potential of UCT MSCs. (**A**) Quantitative RT-PCR analysis showing relative levels of *CD105* mRNA and *CD90* mRNA after transfection of UCT MSCs with siRNAs to human CD105 (KD105), or CD90 (KD90) or a negative control siRNA (Cont) (**p = 0.004, N = 3). Representative western blot analysis of Myogenin (**B)** and MyHC (**C**) in KD105, KD90, or Cont MSCs from UCT that were induced to differentiate into the myogenic lineage after 3 and 5 days respectively. Graphs below show quantitation. Positive controls are human skeletal myoblasts (HM) in (**B**) and primary murine myotubes (MT) in (**C**). β actin was used as loading control (**p = 0.001, N = 3).

To obtain a functional understanding of enhanced myogenic capacities of UCT MSCs over UCB MSC, we addressed whether the differential populations of CD105 and CD90-expressing MSCs within UCT and UCB MSCs were responsible for influencing outcomes in terms of their myogenic potentials. For this, we performed siRNA knockdown assays against CD105 and CD90 in UCT MSCs followed by induction of myogenic differentiation for 3 and 5 days (Fig. 5A N = 3). We observed a significant reduction in relative CD105 and CD90 mRNA levels upon knockdown of their mRNA levels (Fig. 5A). However, western blot analysis revealed a 60% reduction in Myogenin protein after 3 days and a 70% reduction in MyHC protein after 5 days only in CD90, but not CD105 knockdown cells (Fig. 5B,C, full length blots provided in Supplementary Fig. S3). These results suggest that CD90 expression promotes myogenic progression in uMSCs. Importantly, this could explain the enhanced myogenic capacity of UCT MSCs that possess a predominant population of CD105^+^CD90^+^ expressing cells, when compared to UCB MSCs that comprise of CD105^+^CD90^−^ expressing cells. Taken together, our results show that UCT MSCs fundamentally differ from UCB MSCs in terms of key MSC markers, cellular behaviour and myogenic capacity and validate UCT MSCs as a valuable source in therapy and modelling of developmental processes.

## Discussion

Our results demonstrate that MSCs derived from two different compartments of the umbilical cord resulting from term birth pregnancies differ in the combinatorial expression of key surface MSC markers, CD105 and CD90, proliferation potential, senescence characteristics, and their propensity to differentiate into the myogenic lineage using a robust myogenic differentiation protocol. Generation of MSCs with high clinical utilization capability requires a comprehensive understanding of their differentiation potentials to enhance the production of specific cell types. One way to achieve this goal is to characterise appropriate markers that define the multi-potential nature of MSCs under specific growth conditions. In this aspect, several studies have found differences in the presence and function of CD105 and CD90 depending on the cell type, which might influence differentiation potentials as well as transplantation capacities. For instance, high CD90 expression has been associated with increase in the maintenance of the undifferentiated state, such that knocking down CD90 expression increased the osteogenic and chondrogenic potential of MSCs while not altering growth kinetics of the cells^21^. In terms of efficacy for transplantation purposes, decreased positivity of CD90 is human MSCs was associated with a loss of immunosuppressive activity of MSCs resulting in lymphoproliferative allogeneic responses in peripheral blood mononuclear cells^22^. Interestingly, isolation procedures that involved a negative immunodepletion of various antigens prior to plating (CD3, CD14, CD19, CD38, CD66b, and glycophorin A cells) showed an absence of CD90^+^ cells, but a presence of CD105^+^ cells in the resulting MSC populations, suggesting that CD90 expression might be present to a larger extent on CD105^−^ or CD73^−^ populations^23^. Similarly, murine MSCs were found to be heterogeneous for CD105 expression, with CD105- expressing MSCs showing a greater propensity towards tissue differentiation and suppressing the proliferation of CD4T^+^ cells^24^. This suggests that the presence of CD105 like CD90 might function to increase the stemness potential of MSCs with a specific role for CD105, rather than CD90 in proliferation. It is therefore conceivable that the combinatorial presence of CD105 and CD90 would confer increased multi-potentiality and a higher yield in uMSCs. To obtain functional evidence of the role of the two predominant populations of CD105^+^CD90^+^ and CD105^+^CD90^−^ in UCT and UCB respectively in myogenesis, we performed knockdown assays of CD105 and CD90 in UCT MSCs. Unexpectedly, we found that CD90 expression confers increased myogenic potential on UCT MSCs (Fig. 5). This, to our knowledge is the first report that implicates CD90 in regulating myogenic function. In comparison to the demonstration of myogenic differentiation potential by CD105^+^CD31^−^KDR- MSCs from Wharton’s jelly that were induced to differentiate in the presence of a demethylating agent, 5-Azacytidine, our methodology does not use external chromatin modifying agents to induce myogenic differentiation^16^. It is possible that MSCs, in addition to directly influencing myogenic function can also respond to extracellular cues from the milieu. For instance, transplanted MSCs in injured tibialis anterior muscles participated in the muscle recovery process suggesting that the regenerating milieu would have provided essential cues for myogenic lineage determination of MSCs^16^. Indeed, it has been demonstrated that factors secreted from damaged rat skeletal muscle and not intact muscle induce MSCs to transiently express myogenic proteins in a sequential manner^25^. Similarly, MSCs derived from umbilical cord blood were induced to differentiate into muscle by a combinatorial treatment of dexamethasone, hydrocortisone, and 5% horse serum^18^. However, neither of these studies characterised the combinatorial expression of key MSC markers on MSCs vis-à-vis myogenic differentiation, their growth characteristics, and more importantly a comprehensive transcriptomic analysis of conversion into the myogenic lineage. This is especially important given the heterogeneous nature of MSCs isolated from various sources and therefore the need to rigorously define cell surface markers, viability and proliferation features of the population. A key feature in the current study is the detection of Pax7 protein early on during the course of MSC differentiation suggesting the emergence of cells with satellite cell-like properties (Fig. 2A). It remains to be seen whether in fully differentiated cultures, there is a persistence of Pax7+ cells indicative of a self-renewed population. This can be achieved by methods used to isolate “reserve cells”, an in vitro model used to mimic satellite cell behaviour, by trypsinization for brief periods of time to remove differentiated cells leaving behind mononuclear cells that are Pax7 + ve^26^.

A critical component in the comparative analyses between UCB and UCT performed in our studies is the stage of the MSCs at the time of gestation as well as in in vitro cultures. More importantly, comparative analyses were inclusive of paired samples of UCB and UCT MSCs derived from the same neonate for at least 3 of the samples, thus decreasing inter-individual variability. Similar to the increased proliferative potential possessed by younger bone marrow-derived MSCs than older donors, it has been shown that MSCs isolated from foetuses aborted at 8–12 weeks of gestation exhibited more robust proliferative and differentiative potential compared to MSCs isolated from term deliveries at 37–40 weeks of gestation^27–30^. Thus, in our study care was taken to ensure that UCB and UCT MSCs were derived only from one gestational age bracket, i.e., term deliveries. Additionally, during UCT processing, blood vessels (umbilical veins and artery) were removed to ensure there was no contamination from any endothelial MSCs. It is likely that the distribution of CD105^+^CD90^+^ MSCs become separable between fetal tissue compartments with advancing gestational age, such that MSCs with greater multi-potential capacity get restricted to one compartment of the fetal tissue, i.e., the cord tissue.

To obtain an in depth view of the conversion of UCB and UCT MSCs using chemical and adhesion modulation, we performed a transcriptomic analysis of differentiated MSCs after one week of induction of myogenic differentiation (Figs. 3, 4). From these experiments we found that UCT MSCs express greater numbers of myogenic genes that are normally expressed at four-fold higher levels in skeletal muscle compared to other tissues versus UCB MSCs derived from the same neonate (Figs. 3, 4). Additionally, in comparison with a compendium of muscle proteins identified by LC/MS, UCT MSCs express a higher number of genes (red cluster in Fig. 4) compared to UCB MSCs associated with terminal muscle differentiation^20^ (Fig. 4). Regulation of Ceruloplasmin, an enzyme involved in copper transport and iron metabolism and is highly regulated during C2C12 differentiation was found to be the top GO term in the myogenic differentiation of UCT MSCs, highlighting the importance of homeostasis of trace elements during myogenic differentiation of multipotent progenitor stem cells^31^ (Fig. 4). Indeed, a recent study demonstrated that altered regulation of copper during myogenic differentiation might be responsible for the neuromuscular symptoms associated with Menkes and Wilson disease^32^. Genes associated with muscle contractility including myosin isoforms, α-actinin, dystrophin, and troponin were also found to be upregulated in UCT MSCs compared to UCB MSCs further corroborating the robust myogenic differentiation potential of UCT MSCs and the methodology to obtain cells of the myogenic lineage.

## Materials and methods

### Ethics statement

The use of umbilical cord tissue and cord blood in this study was approved by the Institutional Committee for Stem Cell Research (IC-SCR), Institutional Ethics Committee, Translational Health Science and Technology Institute (IEC-THSTI), Institutional Ethics Committee of Civil Hospital, Gurugram, Haryana, and the Institutional Biosafety Committee, THSTI. Human UCB and UCT samples were harvested from term deliveries at the time of birth. Informed written consent was obtained from subjects**.** Information on the participant’s name, age, time of delivery, gestational age at delivery, and weight and sex of the baby were also recorded. All methods were carried out in accordance with relevant guideline and regulations.

### Cord blood, cord tissue collection, and MSC propagation

For UCB samples, 30 ml of blood was collected from the cord vein by venepuncture at the cut end of the cord attached to the placenta in heparin tubes and transported on ice from Gurugram Civil Hospital to THSTI. Samples were processed for MSC isolation within a span of 3 h. For UCB samples, cord blood that was diluted in PBS was layered on Lymphoprep (AXIS SHIELD, cat # 1114547) at a ratio of 2:1 and spun down. Mononuclear cells at the interphase of the diluted plasma and Lymphoprep were carefully collected and seeded in tissue culture plates. For UCT samples, 5 cm of cord tissue was collected and processed within the same time frame as UCB. For preparation of MSCs from cord tissue, the tissue was cut along the longitudinal axis and blood vessels were scraped from the inner surface. The dissection of the cord tissue was performed while it was placed in Phosphate Buffered Saline (PBS) enriched with 5 gm/litre glucose (Sigma Aldrich), 50 ug/ml gentamycin (PAA laboratories), 2.5 ug/ml amphotericin B (Sigma Aldrich), 100 U/ml penicillin and 100ug/ml streptomycin (PAA laboratories). In the laboratory, cord tissue was further minced into 0.5 cm^3^ sized fragments. Minced tissue pieces were placed face down on tissue-culture-treated dishes in media containing MEM Alpha Modification without L-glutamine, ribo- and deoxyribonucleosides (cat # SH30568.FS, Hyclone) and 15% FBS (not heat-inactivated) (Fetal Bovine Serum, qualified, Brazil, cat # 10270106), 50 ug/ml gentamycin in a humidified incubator with 5% CO_2_ at 37 °C temperature. Culture medium was poured gently along the sides to prevent detachment of tissue pieces. Fresh culture medium was added after 3 days. After a week, tissue fragments were removed and culture medium was replaced every 4 days for 1 month until the appearance of adherent cells reaching 80–90% confluency. Adherent cells were harvested using Trypsin/EDTA solution (1X 0.25% Trypsin and 0.02% EDTA in Hanks Balanced Salt Solution (HBSS)). The cell suspension was centrifuged at 1500 r.p.m for 5 min and the cell pellet was resuspended in growth medium. Cells were subcultured at a density of 4000 cells/cm^2^ in a T175 cm^2^ flask and grown till 80% confluence. We observed a difference in the yield of MSCs obtained from UCB samples, with UCB samples generating MSCs along the range of 10^2^ to10^4^, and UCT samples generating MSCs along the range of 10^4^ to10^6^. Further propagation of UCB and UCT derived MSCs was attained by plating in ɑ-MEM (Hyclone, cat # SH30568.FS) with 2 mM sodium pyruvate, 4 mM L-glutamine, 20% FBS, and antibiotics (henceforth referred to as growth medium), with the exception that UCB-derived MSCs were plated in growth medium supplemented with 1 ml of MesenPro growth supplement (ThermoFisher Scientific, cat # 12746012).

### Immunophenotyping of MSCs

To detect surface antigens, cells were detached and washed with phosphate-buffered saline (PBS, TS1101; HiMedia Laboratories, LLC) and incubated at 4 °C for 30 min with the following cell-specific antibodies conjugated to fluorescein isothiocyanate (FITC)- CD90, CD34, and CD45, or phycoerythrin (PE)-CD105, or phycoerythrin-Cy7 (PECy7)-CD73. For FACS analysis to check for hematopoietic lineage cells, MSCs were stained for CD105-PE and CD45-FITC and CD34-FITC. Labelled cells were analysed by Flow cytometry (FACSCanto II or FACSAria III, BD Biosciences, San Jose, CA). FlowJo software (TreeStar, Ashland, OR) was used for analysis.

### Myogenic differentiation of MSCs

Myogenic differentiation was induced by culturing MSCs in tissue culture plates coated with 0.01% Collagen Type 1 (Merck cat # C8919) and 20 ug/ml Laminin (Merck, cat # L2020) at a density of 10,000 cells/cm^2^ in growth medium. After reaching a confluence of 70%, myogenic differentiation was induced by adding M1 medium (DMEM, 5% horse serum, 0.1 µM dexamethasone, and 50 µM hydrocortisone) or M2 medium (DMEM, 15% KnockOut Serum Replacement (KSR, ThermoFisher Scientific), 0.5% DMSO (Merck), CHIRON99021 (Tocris) at 1 μM and 0.1 μM LDN193189 (Tocris) for 5 days followed by DMEM, 15% KSR, 0.1% BSA supplemented with 10 ng/ml HGF, 2 ng/ml IGF-1, 20 ng/ml FGF-2 (Peprotech, Merck) and 0.1 μM LDN193189 for the rest of the incubation period. For comparative analysis of myogenic differentiation between M1 and M2 media, media was changed every other day. We performed 3 technical replicates for each of the 5 biological donors for UCB and UCT.

### Senescence and proliferation assays

Senescence in UCT and UCB MSCs was determined using the Senescence β-galactosidase staining kit (cat # 9860, Cell Signaling Technology) and performed as per the manufacturer’s instructions. Briefly, UCT and UCB MSCs from passages P6 were plated at a density of 2 × 10^5^ cells/well in growth medium. Cells were fixed in for 15 min at room temperature followed by staining with β-galactosidase staining solution, sealed and stored at 37 °C in a dry incubator. Cells were periodically checked for staining and reaction was stopped at the same time in both UCB and UCT MSC seeded plates and processed for microscopic analysis. For assessment of proliferation, 2 µM EdU (5-ethynyl-2′-deoxyuridine) was added to UCB and UCT MSCs from P4 passage for 6 h and fixed and stained for EdU using the Click-iT EdU cell proliferation kit (cat # 10337, ThermoFisher Scientific). Cells were then counterstained with DAPI to visualize nuclei.

### RNA-sequencing analysis

RNA-sequencing analysis was performed on MSCs obtained from UCB and UCT from a single neonate with duplicate technical replicates for undifferentiated and differentiated MSCs. Cells were washed in PBS on ice and RNA was extracted with Trizol (Invitrogen). RNA purification was done using the Direct-zol TM RNA MiniPrep kit (catalog # R2050, Zymo Research). Purified RNA samples were sent to Bencos Research Solutions for analysis. High throughput RNA seq analysis was performed on cell lysates using Illumina HiSeq 4000/Novaseq 6000. The quality assessment was carried out on raw Fastq files received using NGS QC toolkit with a minimum average quality (Q) value of 30 and an overall high quality read mapping set at 70% of 150 bp DNA fragment size. Filtered reads were mapped to reference genome (GRCh38) using STAR alignment tool^33^. Read counts per gene was calculated using HTSeq-count^34^. After quantification Deseq2 was used for differential gene expression analysis^35^. Heatmap for Fig. 4A was created using seaborn clustermap function. Normalized read counts for the genes coding for known skeletal muscle proteome was used as the input data. ‘Ward’s method was used for clustering. For GO analysis, Metascape tool was used^36^.

### siRNA transfection assays

UCT MSCs from their 3rd passage in culture were plated in 6-well plates at a density of 2 × 10^5^ cells/well in growth medium and allowed to attach overnight. The cells were then transfected with pool siRNAs to CD105 (Silencer Pre-designed siRNA, Assay ID 145526 and Assay ID 7906, cat # AM16708, ThermoFisher Scientific) or CD90 (Silencer Pre-designed siRNA, Assay ID s14125, cat # 4392420, ThermoFisher Scientific). Control wells were transfected with a Silencer negative control siRNA (cat # AM4613, Thermo Fisher Scientific). Transfection was performed using Fugene 6 Transfection reagent (cat # 11814443001, Roche) as per manufacturer’s instructions. Lipid-siRNA complexes were incubated in Opti-MEM Reduced Serum Medium (cat # 31985070, ThermoFisher Scientific) for half an hour and was then added to MSCs in growth medium. After 48 h, myogenic differentiation was induced by replacing transfection medium with M1 medium. At least 3 biological replicates were performed for each knockdown assay.

### Quantitative RT-PCR

Quantitative RT-PCR was performed using the CFX96 Touch RT-PCR Detection System (Bio-Rad Laboratories Inc.) and SsoFast EvaGreen Supermix (Cat # 172-5204AP, Bio-Rad Laboratories Inc.). Each sample was amplified in triplicate with published human primers specific to *Pax7*^37^, *Myf-5*^37^, *MyoD*^38^, *Myogenin*^39^, *MyHC*^40^, *CD105*^41^, and *CD90*^41^. Expression levels were normalized *Gapdh.*

### Statistical methods

For all cell culture experiments involving MSC characterization, 5 biological replicates (N) along with 3 technical replicates (n) for each of the biological replicates. Data are represented as mean ± standard error of the mean (SEM). Normalized distribution of data was observed prior to conducting two-tailed Students *t*-tests to test for statistically significant differences between groups using GraphPad Prism software. Differences were considered significant at the *p* < 0.05 level.

## Supplementary information

Supplementary Information.

## Data Availability

The RNA-Seq data used to support this study is uploaded to NCBI, the SRA accession is GSE147114.
