# Publisher Correction: Umbilical cord tissue is a robust source for mesenchymal stem cells with enhanced myogenic differentiation potential compared to cord blood

**DOI:** 10.1038/s41598-021-93047-5

**Published:** 2021-06-29

**Authors:** Shivangi Mishra, Jayesh Kumar Sevak, Anamica Das, G. Aneeshkumar Arimbasseri, Shinjini Bhatnagar, Suchitra D. Gopinath

**Affiliations:** 1 Pediatric Biology Center, Translational Health Science and Technology Institute (THSTI), NCR Biotech Science Cluster, 3rd Milestone, Faridabad-Gurgaon Expressway, PO Box #04, Faridabad, 121001 India; 2 National Institute of Immunology, Aruna Asaf Ali Marg, New Delhi, India

Correction to: *Scientific Reports* 10.1038/s41598-020-75102-9, 04 November 2020

The Supplementary Information file published with this Article contained an error where the Supplementary Figures were omitted. The original Supplementary Information file is provided below

This error has now been corrected in the Supplementary Information file that accompanies the original Article.

## Supplementary Information

Supplementary Information.

