# Supplementary material for: Umbilical cord tissue is a robust source for mesenchymal stem cells with enhanced myogenic differentiation potential compared to cord blood
Source: Sci Rep. 2020 Nov 4;10:18978. doi: 10.1038/s41598-020-75102-9 (PMC7642376; doi:10.1038/s41598-020-75102-9)
Supplement: Supplementary file 1 — Supplementary Information. [file 41598_2020_75102_MOESM1_ESM.pdf]

Umbilical cord tissue is a robust source for mesenchymal stem cells with enhanced myogenic differentiation potential compared to cord blood

Running title: Myogenic differentiation and mesenchymal stem cells

Shivangi Mishra[1#], Jayesh Kumar Sevak[1#], Anamica Das[2], G Aneeshkumar Arimbasseri [2], Shinjini Bhatnagar[1], and Suchitra D. Gopinath[1*]

Material and Methods

Immunocytochemistry and Flow Cytometry

MSCs incubated in myogenic differentiation medium M1 for 3, 7,15, or 21 days were washed twice with cold PBS and fixed with 4% paraformaldehyde for 15 minutes. For detection of myogenic proteins by FACS, cells were detached after respective time points and fixed with ice cold methanol at -20^{0}C for 10 minutes. Permeabilization buffer (Tris-buffered saline, 0.1% Triton X-100) was added to the cells for 15 minutes followed by incubation with blocking buffer (permeabilization buffer with 2% horse serum) for an hour at room temperature. Cells were incubated with primary antibodies to anti-Pax7 (1:50, DHSB), anti-MyoD (1:100, Novus Biologicals), anti-Myogenin (1:50, Clone F5D, Santa Cruz Biotechnology), and anti-MyHC (1:50, My-32, Sigma) antibodies overnight at 4^{0}C followed by incubation with secondary antibody, anti-mouse Alexa Fluor 488 (Invitrogen). To detect nuclei, cells were counterstained with 4',6-diamidino-2-phenylindole (DAPI).

Western Blotting

Protein extracts from MSCs were obtained by lysing cells in lysis buffer (50 mM Tris-HCl pH 7.5, 150 mM NaCl, 5 mM EDTA, 1% NP40, 0.5% sodium deoxycholate, 0.1% SDS). The proteins were resolved by SDS-PAGE (8%) and transferred onto nitrocellulose membranes. The membranes were incubated with primary antibodies followed by incubation with HRP-conjugated anti-mouse or anti-rabbit secondary antibodies and visualized using SupersignalWest Pico ChemiluminescentSubstrate (Thermo Scientific, Rockford, IL, USA).

Supplementary Figure legends

S1. (A) Expression of myogenic proteins in C2C12 cells: Proliferating C2C12 cells and cultures differentiated for 2 days and 4 days in the presence of 2% horse serum were fixed and stained for Pax7 and MyoD and Myogenin and MyHC respectively and analyzed by flow cytometry. This was used as a control group for MSCs. (**B**) **Comparison of myogenic capacities between UCT and UCB MSCs:** UCT and UCB MSCs induced to differentiate for 10 days in M1 media were stained for MyHC. Both UCT and UCB MSCs stained positive for MyHC showing different morphologies, with elongated cells in UCT MSCs and myosacs in UCB MSCs.

S2. Full length blots for Myogenin and GAPDH. Bordered area shows band of interest.

S3. Full length blots showing Myogenin (left) and MyHC (right) and β actin (below) in Cont, KD105, and KD90 samples. Line shows band of interest with respect to the protein molecular weight marker.

S1

A

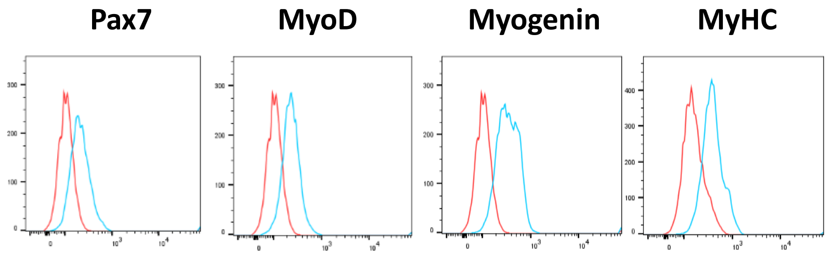

B

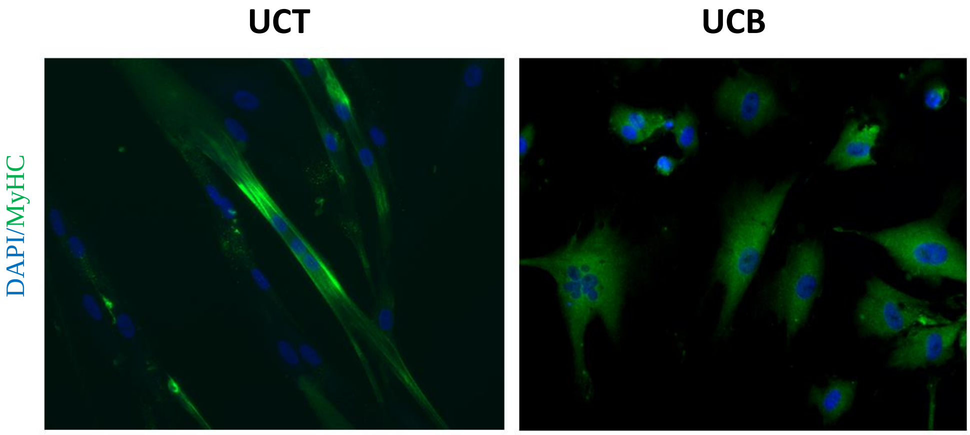

S2

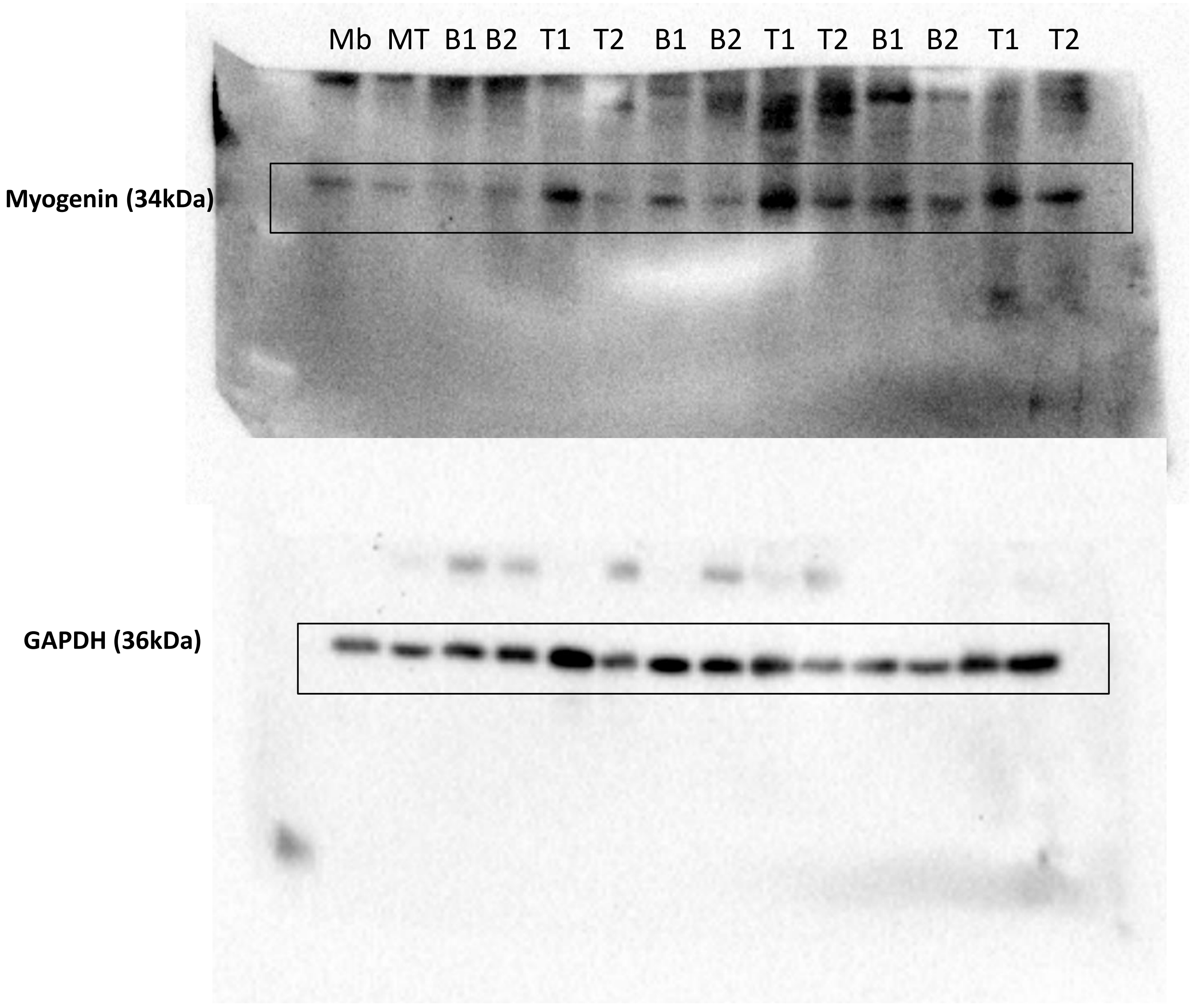

Mb MT B1 B2 T1 T2 B1 B2 T1 T2 B1 B2 T1 T2
Myogenin (34kDa)
GAPDH (36kDa)

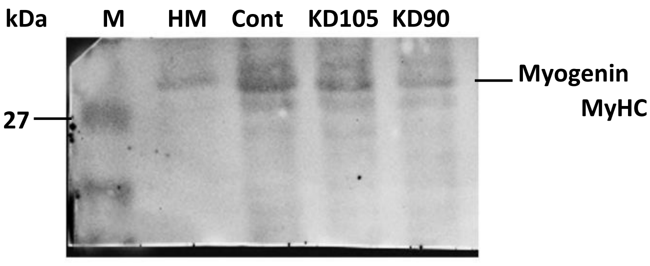
kDa
M
HM
Cont
KD105
KD90
Myogenin
27

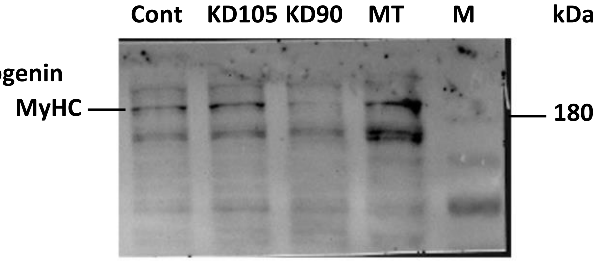
Cont
KD105
KD90
MT
M
kDa
MyHC
180

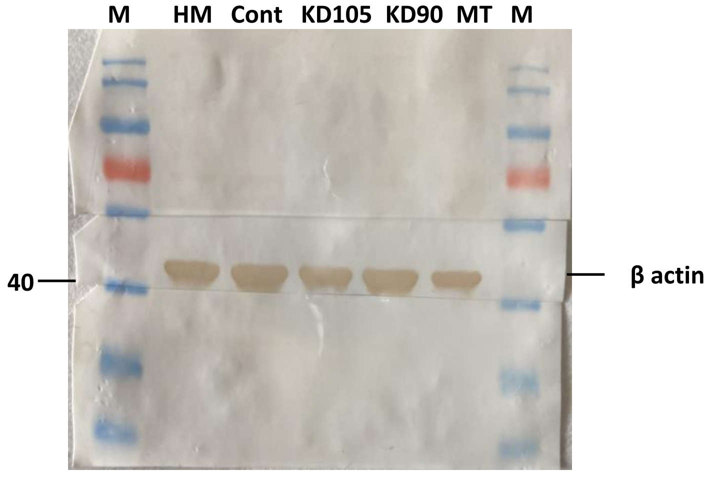
M
HM
Cont
KD105
KD90
MT
M
40
β actin
